# Effectiveness and economic evaluation of chiropractic care for the treatment of low back pain: a systematic review protocol

**DOI:** 10.1186/s13643-015-0015-5

**Published:** 2015-03-18

**Authors:** Marc-André Blanchette, André Bussières, Mette Jensen Stochkendahl, Jill Boruff, Pamela Harrison

**Affiliations:** Public Health PhD Program, School of Public Health, University of Montreal, 7101 Parc avenue, Montreal, QC H3N 1X9 Canada; School of Physical and Occupational Therapy, Faculty of Medicine, McGill University, 3654 prom Sir-William-Osler, Montreal, QC H3G 1Y5 Canada; Centre de Recherche Interdisciplinaire en Réadaptation de Montréal, 7005 Maisonneuve Boulevard West, Montreal, QC H4B 1 T3 Canada; Département chiropratique, Université du Québec à Trois-Rivières, 3351 Des Forges Boulevard, Trois-Rivières, QC G9A 5H7 Canada; Nordic Institute of Chiropractic and Clinical Biomechanics, Campusvej 55, DK-5230 Odense M, Denmark; McGill University, 809 Sherbrooke Street West, Montreal, QC H3A 0C1 Canada

**Keywords:** Low back pain, Systematic review, Chiropractic, Economic evaluation

## Abstract

**Background:**

Chiropractic care is a common treatment for low back pain (LBP). Previous studies have failed to clarify the relative cost-effectiveness of chiropractic care in comparison with other commonly used approaches because previous attempts to synthetize the economic literature has only included partial economic evaluations. The objective of this project is to estimate the clinical effectiveness and cost-effectiveness of chiropractic care compared to other commonly used care approaches among adult patients with non-specific LBP.

**Methods/design:**

Two systematic reviews will be conducted to identify 1) randomized controlled trials and 2) full economic evaluations of chiropractic care for low back pain compared to standard care provided by other healthcare providers. We will conduct searches in specialized electronic databases for randomized controlled trials and full economic evaluations published between 1990 and 2014 using a combination of keywords and MeSH terms. This will be supplemented by a search of the gray literature. Citations, abstracts, and relevant papers will be screened for eligibility by two reviewers independently. Studies will be critically appraised using 1) the Cochrane risk of bias tool and 2) the Drummond (BMJ) checklist. Results will be summarized using Slavin’s qualitative best-evidence synthesis approach. Data relating to the primary outcomes of the effectiveness study will be evaluated for inclusion in meta-analyses. The costs will be standardized to the same currency (USD) and adjusted to the same year for inflation. The incremental cost-effectiveness, incremental net benefit, and relevant confidant intervals will be recalculated in order to facilitate comparison between studies.

**Discussion:**

Our review will evaluate both the clinical effectiveness and the cost-effectiveness associated with chiropractic care for LBP. A more precise estimate of the cost-effectiveness of chiropractic care for LBP relative to other forms of conservative care is needed for decision-makers and third-party payers to offer best care options for LBP. Our results will facilitate evidence-based management of patients with LBP and identify key areas for future research.

**Systematic review registration:**

The protocol is registered on PROSPERO (CRD42014008746).

**Electronic supplementary material:**

The online version of this article (doi:10.1186/s13643-015-0015-5) contains supplementary material, which is available to authorized users.

## Background

Low back pain (LBP) remains a leading cause of disability worldwide, accounting for over 10% of the total of ‘years lived with disability’ [1]. LBP is the most common occupational injury in Canada and United States [2,3]. It is the leading cause of work absenteeism and ranks sixth among health problems in terms of direct medical costs in North America [4].

The incidence of non-specific LBP has not significantly increased in the last four decades [5-7]. However, a drastic increase in the number of certificates of illness and benefits paid for chronic disabilities resulting from LBP has been reported in industrialized countries since the 1980s [8]. Such increase in disability level has had an alarming impact on costs due to lost productivity, wage replacement, and health care utilization. According to the 2010 Global Burden of Disease Study, low back pain disability-adjusted life years increased from 58.2 million in 1990 to 83.0 million in 2010 [9]. With the hope of reducing the significant health and economic burden associated with LBP, researchers have examined the effectiveness of numerous treatment options, including manual therapy [10-14].

Opinions vary widely on what causes LBP and how best to manage it [15]. It is estimated that over 85% of patients with LBP have symptoms that are ‘non-specific’ in nature since they cannot reliably be attributed to a specific disease or anatomical structure [16]. Perhaps as a result, relatively few treatment modalities for the management of LBP have been shown to achieve superior and sustained improvements in pain, physical function, and disability [17,18]. An example is spinal manipulative therapy (SMT), which recent reviews did not find significantly more effective than and other modalities [11,19,20]. SMT is often a core component of chiropractic care [21], but chiropractic care is not restricted to the use of SMT [22], and a range of other treatment modalities may be offered exclusively or in combination with SMT to potentially compliment or enhance treatment outcome. Moreover, SMT is not performed exclusively by chiropractors but is used extensively worldwide by a range of other health care professionals [11,20]. In the case of SMT, studies on the effectiveness may guide clinicians (chiropractors and others) in their choice of treatment modality, but offer little information to patients, policy makers, and third-party payers about the clinical effectiveness and cost-effectiveness of standard care offered by different providers.

Considering this, older reports [23,24] have pointed to chiropractic care as efficient for the treatment of LBP because of the relatively low fee for service, the use of ‘low tech’ therapies such as manual therapy, and the low usage of costly investigations such as advanced diagnostic imaging. However, when compared with medical and physiotherapy care, economic reviews have not been able to support this or provide clear guidance to informed decision-making regarding the many available provider options [25,26]. This divergence in findings may be partly due to the limited number of studies of acceptable methodological quality [25,27] and partly because the previous systematic reviews have only included partial economic evaluation (cost description, cost analysis, and cost-outcome description). The last systematic review of economic and clinical effectiveness studies of chiropractic care was completed nearly a decade ago and provided limited guidance [25]. We are conducting this review with the hope of including more high quality studies.

When evaluating standard care for LBP, decisions to recommend any one option should preferably be based on the clinical effectiveness, the cost-effectiveness, the safety of the approach, and patient preference [11,19,26]. Only full economic evaluation (cost-effectiveness analysis, cost-utility analysis, cost-benefit analysis) of standard care practice can provide adequate information about resource inputs (costs) and outputs (health outcomes) [28] and evaluate whether healthcare resources are being used optimally [29]. In order to better inform patients, policy makers, and third-party payers about the clinical effectiveness and cost-effectiveness of standard chiropractic care for LBP in comparison to usual standard care provided by other health care professionals, an evidence synthesis is indicated.

### Objectives

The two main objectives of this review are 1) to estimate the extent to which chiropractic care is effective for adult patients with non-specific low back pain compared to other conservative care approaches (for example, medical care and physiotherapy) and 2) to estimate the cost-effectiveness of chiropractic care for adult patients with non-specific LBP compared with other conservative care approaches.

## Methods/design

### Eligibility criteria

To be eligible for inclusion, studies must meet the following criteria:

1. The study design is:
  a randomized controlled trial for the clinical effectiveness studies;
  a full economic evaluation (including cost-effectiveness, cost-utility, cost-benefit analyses, and cost-minimization analysis alongside a clinical trial [30]) for the economic studies.
2. The population under study is composed of adult patients (≥18 years) with non-specific LBP with or without sciatica of any duration. Studies reporting multiple pain locations or spinal pain without separate results for LBP will be excluded.
3. The intervention is chiropractic care. Studies that evaluate chiropractic care as part of a combined, multidisciplinary approach will be excluded unless the chiropractic care part is evaluated separately. Studies that evaluate specific treatment modalities (for example, SMT) will be excluded.
4. The comparator is non-surgical, usual conservative care delivered by other healthcare providers (for example, medical therapy, physical therapy, or acupuncture). Studies including surgical treatment of LBP as the only comparator will be excluded.
5. The outcome must include - for the clinical effectiveness studies - one or more of the following primary or secondary effect measures:

### Primary outcomes

- Pain (for example, visual analog scale, numerical rating scale, McGill pain score)
- Functional status (for example, Roland-Morris questionnaire, Oswestry Disability Index)
- Global improvement (for example, the number of patients reporting to have recovered)

### Secondary outcomes

- Health related quality of life (for example, SF-36, EuroQol)
- Return to work
- Adverse effects

–For the economic studies: an incremental measure of the extra budget required to improve an additional unit of outcome (that is, an incremental cost-effectiveness ratio or an incremental net benefit measure) with the exception of cost-minimization studies.

6. Studies must be published in English or French.
7. Studies without full-text manuscript available (for example, abstracts, conference proceedings, presentations) and duplicate study reports will be excluded. Published study protocol will be registered but not included in the data analysis.

### Information sources and search

A comprehensive literature search will be conducted using indexed subject headings and free text related to the topic of interest in electronic health literature databases, as well as gray literature sources (economic evaluations only), to uncover potentially relevant studies. With the exception of PubMed, the search will be limited to studies published between 1990 and the search date. Since the volume of literature on back pain is impressive, we restricted search to 1990 (start date) as it corresponds with the first potentially relevant studies in this topic. This start date will enable us to select relatively recent literature that is compatible with the contemporary practice of both chiropractic and comparator providers. The PubMed search will be used to retrieve the most recent publications and restricted to items published on or after 2014.

Searches will be conducted in the following electronic databases: Ovid Medline, Ovid AMED, Ovid EMBASE, CINAHL, the Cochrane Database of Systematic Reviews, and PubMed. In addition, we will also search for economic evaluations in the following: Index to Chiropractic Literature (ICL), Cochrane Library, Health Technology Assessment Database, and ECONLIT. Finally, a search of the gray literature for economic evaluations will include the websites of the following organization: Canadian Institute for Health Information (CIHI), Canadian Agency for Drugs and Technologies in Health (CADTH), Canadian Institute of Health Research (CIHR), Tufts Medical Center Cost-effectiveness Analysis Registry, Agency for Healthcare Research and Quality, National Institute for Health Research Health Technology Assessment program, and National Institute for Health and Care Excellence (NICE).

The search strategy will be different for each database, and the RCT filters for PubMed, Ovid Medline, AMED, EMBASE, and CINAHL will be adapted from the Cochrane Highly Sensitive Search Strategy for identifying randomized trials in MEDLINE [31]. The search in Cochrane will be limited to Cochrane Central (Trials) to exclude other study designs. Two clinical librarians (JB and PH), with experience in searching for systematic reviews, developed a search strategy for each individual database and will conduct the searches. The search strategy for clinical effectiveness and for cost-effectiveness can be found in Appendix 1 and 2, respectively. We will screen the bibliographies of relevant publications, including reviews and meta-analyses, for additional relevant articles.

### Study selection

Titles and abstracts of studies identified from the literature search will be combined using Endnote 14 and screened for relevance by two independent reviewers to identify all articles that any reviewer judges potentially eligible. The same reviewers will independently apply eligibility criteria to the full-text manuscript of all potentially eligible studies. Disagreements will be discussed until consensus. Disagreements will be resolved with arbitration by a third reviewer if disagreements persist.

### Quality assessment and analysis

All eligible studies on clinical effectiveness will be assessed for methodological quality (risk of bias) by two independent reviewers. Studies assessing clinical effectiveness will be evaluated using 12 criteria recommended by the Cochrane Back Review Group [32]. These criteria include blinding of the patient, treatment provider, and outcomes assessor. Studies that meet at least 6 criteria out of 12 will be considered at low risk of bias, while the others will be considered at high risk of bias.

Studies assessing costs will be evaluated using a recommended tool for health economic evaluations, the Drummond (BMJ) checklist [29,33,34]. This checklist includes 35 items grouped into four broad categories: general issues about study design, data collection, data analysis, and interpretation of results. Any disagreements between reviewers will be discussed until consensus is reached or with arbitration by a third reviewer if disagreements persist. The quality level (low, medium, high) of every study will be determined by agreement between three investigators (AB, MAB, MJS). This will enable the investigators to formulate a qualitative appreciation of the complete study.

### Data extraction

Data will be extracted separately by two independent reviewers; any disagreements will be resolved through discussion, with arbitration by a third reviewer if necessary. Authors of potentially relevant studies will be contacted regarding additional information or missing data. Key findings from each study will be summarized and presented in a summary tables. Two separate forms will be used for clinical effectiveness studies and economic evaluations. For clinical effectiveness studies, we will use the Cochrane back review group data extraction form [35]. Extracted variables will include author and year; country; participants, indication, setting; compared treatments; time horizon, outcomes assessed; authors’ results; and conclusion.

Data from economic evaluations will be extracted using a customized data extraction sheet (Additional file 1). Extracted variables will include author and year; country; type of economic evaluation; participants, indication, setting; compared treatments; perspective; time horizon, currency price (year); included costs, health effect (pain, functional status, global improvement, health-related quality of life, return to work); mean costs, mean quality-adjusted life years (QALYs); incremental cost-effectiveness statistics; limitations; and authors’ conclusion.

### Measures of effect estimates

Continuous outcomes measured with the same instrument (that is, pain measured with visual analog scale) will be compared using mean difference, whereas continuous outcomes measured with different instruments (that is, functional status measured with Roland-Morris or Oswestry tools) will be compared using standardized mean difference. For dichotomous outcomes (that is, recovery, return-to-work), a risk ratio will be generated.

### Data analysis

Effect measures relating to the primary and secondary outcomes of clinical effectiveness studies with low risk of bias, and no serious flaw will be evaluated for inclusion in the meta-analyses. Outcomes will be assessed at 1, 3, and 12 months and will be categorized according to the time closest to these intervals. In order to minimize clinical diversity, we will stratify by healthcare provider (for example, chiropractic care versus medical care or chiropractic care versus physiotherapy), symptom duration (acute (0 to 6 weeks), sub-acute (6 to 12 weeks), chronic (more than 12 weeks), and mixed/not specified), and outcomes (type of outcome and time of assessment). Heterogeneity will be investigated by subjective interpretation and by statistical testing using the *Q* and *I*^2^ test. A cutoff of 40% at the *I*^2^ test will determine the limit of acceptable heterogeneity. If the *I*^2^ cutoff is exceeded or the description of the average care provided by the comparator seems too heterogeneous, results will be discussed narratively in the manuscript without pooled estimates. A sensitivity analysis will be performed by including studies with high risk of bias. Funnel plots will be constructed using all data from the primary outcomes regardless of the comparator or follow-up interval in order to evaluate possible publication bias.

For the economic evaluations, the difference in perspective of analysis, type of economic analysis, and healthcare system will be discussed narratively. To allow direct comparisons across countries and years, we will convert reported costs estimates to 2014 United States (US) dollars. International exchange rate based on purchasing power parities (PPP) will be use to convert cost estimates to US dollars, and gross domestic product (GDP) deflators will be use to convert cost estimates to 2014. PPP and GDP are available from the World Economic Outlook Database (http://www.imf.org/external/data.htm). Results comparing chiropractic to other types of care will be summarized using Slavin’s [36] qualitative best-evidence synthesis approach, which assumes that the strength of a relationship between variables is based on the quantity and quality of the evidence available. This approach aims to provide methodological rigor by clearly and concisely articulating the synthesis criteria and was recently used in a number of systematic reviews related to occupational health [37-39]. The level of evidence uncovered for the findings of interest will be assessed using a 5-point ordinal scale (strong, moderate, limited, mixed, and insufficient evidences) defined by Slavin [36]. The appropriate level of evidence for each finding will be assessed in a stepwise manner by first determining if criteria for the highest level of evidence (that is, strong) are fulfilled and, if they are, no further evaluation is performed. If those criteria are not fulfilled, those for the next lowest level of evidence are then assessed, continuing until the appropriate level of evidence can be assigned to the various review findings. The criteria for each level of evidence are the following:

#### Strong evidence

Minimum of three high quality studies; at least three quarters of high and medium quality studies must concur on findings.

#### Moderate evidence

Minimum of two high quality studies or three of medium and high quality; more than two thirds of all studies must report consistent findings.

#### Limited evidence

Minimum of one high quality study or two medium quality studies, more than 50% of all studies must report consistent findings.

#### Mixed evidence

Findings from medium and high quality studies are contradictory.

#### Insufficient/no evidence

No high quality studies; one or no medium quality studies; any number of low quality studies.

### Protocol registration

Our protocol is registered on PROSPERO (CRD42014008746), http://www.crd.york.ac.uk/PROSPERO. This manuscript conforms to the PRISMA guidelines [40] that are relevant to the reporting of a systematic review protocol. We present our methods and analysis for the review of clinical effectiveness and our review of economic evaluations separately.

## Discussion

Our research team includes French and English investigators. The potential of omitting important studies in other languages is considered very small since chiropractic is of English/American origin and is primarily practiced in the anglophone countries.

Decisions regarding optimal care should be based on aspects of importance to all stakeholders, including clinical effectiveness, harms, patient preference, and cost-effectiveness. A more precise estimate of the cost-effectiveness of chiropractic care for LBP relative to other forms of conservative care is needed for decision-makers and third-party payers to offer best care options for LBP. Evidence is also needed to help guide employer and regulatory decisions to reduce unnecessary costs for work-related LBP resulting in temporary or permanent disability [18].

## Appendix 1: Search strategy for the review of clinical effectiveness

**PUBMED**

**Limit: publication dates: from 2014/03/21 -**

publisher[sb]

**AND**

(

“back pain”[tiab] OR

backache[tiab] OR

“spine pain”[tiab] OR

“spinal pain”[tiab] OR

(“back disorder”[tiab] OR “back disorders”[tiab]) OR

(sciatic[tiab] OR sciatica[tiab]) OR

ischialgia[tiab] OR

((degenerate[tiab] OR degeneration[tiab] OR degenerating[tiab] OR degenerated[tiab] OR prolapse[tiab] OR prolapsing[tiab] OR prolapsed[tiab] OR hernia[tiab] OR hernias[tiab] OR herniating[tiab] OR herniate[tiab] OR herniated[tiab] OR bulge[tiab] OR bulges[tiab] OR bulging[tiab] OR bulged[tiab] OR protrude[tiab] OR protruding[tiab] OR protruded[tiab] OR protrusion[tiab] OR protrusions[tiab] OR extrude[tiab] OR extruding[tiab] OR extruded[tiab] OR extrusion[tiab] OR extrusions[tiab] OR sequestrate[tiab] OR sequestrated[tiab] OR sequestrating[tiab] OR sequestration[tiab] OR sequestrations[tiab] OR disorder[tiab] OR disorders[tiab] OR disordered[tiab] OR disease[tiab] OR diseases[tiab] OR diseased[tiab] OR rupture[tiab] OR ruptures[tiab] OR ruptured[tiab] OR slip[tiab] OR slips[tiab] OR slipped[tiab]) AND (disc[tiab] OR discs[tiab] OR discal[tiab] OR disk[tiab] OR disks[tiab])) OR

(“spinal stenosis"[tiab] OR “spinal stenoses”[tiab]) OR

(“lumbar stenosis”[tiab] OR “lumbar stenoses”[tiab)] OR

(discitis[tiab] OR discitides[tiab] or diskitis[tiab] OR diskitides[tiab] OR spondylodiskitis[tiab] OR spondylodiskitides[tiab] OR spondylodiscitis[tiab] OR spondylodiscitides[tiab]) OR

(“vertebrogenic pain syndrome”[tiab] OR “vertebrogenic pain syndromes”[tiab]) OR

(zygapophyseal[tiab] OR “facet joint”[tiab]) OR “facet joints”[tiab]) OR

lumbar[tiab] OR

lumbago[tiab] OR

dorsalgia[tiab]

)

**AND**

(

(spinal[tiab] OR lumbar[tiab] OR cervical[tiab]) AND manipulation[tiab]) OR

(chiropractic[tiab] OR chiropraxic[tiab]) OR

((back[tiab] OR spine[tiab] OR spinal[tiab] OR lumbar[tiab] OR musculoskeletal[tiab]) AND (adjustment[tiab] OR adjustments[tiab] OR adjusting[tiab] OR adjust[tiab] OR adjusted[tiab] OR manipulation[tiab] OR manipulations[tiab] OR manipulating[tiab] OR manipulate[tiab] OR manipulated[tiab] or mobilization[tiab] or mobilisation[tiab])) OR

(“musculoskeletal manipulation”[tiab] OR “musculoskeletal manipulations”[tiab] OR “manual therapy”[tiab] OR “manual therapies”[tiab] OR “manipulation therapy”[tiab] OR “manipulation therapies”[tiab] “manipulative therapy”[tiab] OR “manipulative therapies”[tiab]) OR

(manipulation[tiab] AND medicine[tiab]) OR (manipulations[tiab] AND medicine[tiab]) OR

flexion[tiab] OR

(“myofascial release”[tiab] OR “myofascial releases”[tiab] OR “myofascial therapy”[tiab] OR “myofascial therapies”[tiab]) OR

(“muscle energy technique”[tiab] OR “muscle energy techniques”[tiab]) OR

(“trigger point”[tiab] OR “trigger points”[tiab] OR “trigger area”[tiab] OR “trigger areas”[tiab]) OR

"Proprioceptive Neuromuscular Facilitation"[tiab] OR

"Cyriax Friction"[tiab] OR

"strain counterstrain"[tiab] OR

(craniosacral[tiab] OR "cranio sacral"[tiab]) OR

("complementary therapy”[tiab] OR “complementary therapies”[tiab] OR “alternative therapy”[tiab] OR “alternative therapies”[tiab] OR “alternative medicine”[tiab] OR “alternative medicines”[tiab])

)

**AND**

(

randomized[tiab] OR

placebo[tiab] OR

“drug therapy”[tiab] OR

“drug therapies”[tiab] OR

randomly[tiab] OR

trial[tiab] OR

groups[tiab]

)

**MEDLINE**

**Database: Ovid MEDLINE(R) In-Process & Other Non-Indexed Citations, Ovid MEDLINE(R) Daily, Ovid MEDLINE(R) and Ovid OLDMEDLINE(R) 1946 to Present**

1. exp Back Pain/
2. back pain.tw.
3. backache*.tw.
4. Sciatica/
5. Sciatica.tw.
6. (spine pain or spinal pain).tw.
7. back disorder*.tw.
8. (Sciatic adj3 (Neuralgia or Bilateral)).ti,ab.
9. ischialgia.tw.
10. ((disc* or disk*) adj3 (degener* or displace* or prolaps* or hernia* or bulge or protrusion* or extrusion* or sequestration* or disorder* or disease* or rupture* or slipped)).tw.
11. Spinal Stenosis/
12. ((stenosis or stenoses) adj3 (lumbar or spine or spines or spinal)).tw.
13. Discitis/
14. diskitis.ti,ab.
15. spondylodiscitis.ti,ab.
16. vertebrogenic pain syndrome*.tw.
17. ((Zygapophyseal or Facet or facets) adj3 (syndrome* or degenerat*)).tw.
18. (lumbar adj3 (ache* or pain* or strain*)).tw.
19. lumbar vertebrae.mp.
20. (lumbago or dorsalgia).tw.
21. 1 or 2 or 3 or 4 or 5 or 6 or 7 or 8 or 9 or 10 or 11 or 12 or 13 or 14 or 15 or 16 or 17 or 18 or 19 or 20
22. exp spinal manipulation/
23. manipulation, chiropractic/
24. chiropractic/
25. chiropract*.tw.
26. ((back or spine or spinal or lumbar or musculoskeletal) adj3 (adjust* or manipulat* or mobili*ation)).tw.
27. exp Musculoskeletal Manipulations/
28. manual therap*.tw.
29. (Manipulati* adj (therap* or medicine)).tw.
30. (Flexion adj2 distraction*).tw.
31. (myofascial adj3 (release or therap*)).tw.
32. Muscle energy technique*.tw.
33. Trigger point*.tw.
34. Trigger Points/
35. Proprioceptive Neuromuscular Facilitation.tw.
36. Cyriax Friction.tw.
37. (Strain adj counterstrain).tw.
38. (Craniosacral Therap* or Cranio sacral Therap*).tw.
39. Complementary Therapies/
40. or/22-39
41. randomized controlled trial.pt.
42. clinical trial.pt.
43. randomi?ed.ti,ab.
44. placebo.ti,ab.
45. randomly.ti,ab.
46. trial.ti,ab.
47. groups.ti,ab.
48. or/41-47
49. animals/
50. humans/
51. 49 not (49 and 50)
52. 48 not 51
53. 21 and 40 and 52
54. limit53 to yr=”1990-Current”

**AMED**

**Database: AMED (Allied & Complementary Medicine) 1985-April 2014**

1. exp backache/
2. back pain.tw.
3. backache*.tw.
4. Sciatica/
5. Sciatica.tw.
6. (spine pain or spinal pain).tw.
7. back disorder*.tw.
8. (Sciatic adj3 (Neuralgia or Bilateral)).ti,ab.
9. ischialgia.tw.
10. ((disc* or disk*) adj3 (degener* or displace* or prolaps* or hernia* or bulge or protrusion* or extrusion* or sequestration* or disorder* or disease* or rupture* or slipped)).tw.
11. Spinal Stenosis/
12. ((stenosis or stenoses) adj3 (lumbar or spine or spines or spinal)).tw.
13. diskitis.ti,ab.
14. spondylodiscitis.ti,ab.
15. vertebrogenic pain syndrome*.tw.
16. ((Zygapophyseal or Facet or facets) adj3 (syndrome* or degenerat*)).tw.
17. (lumbar adj3 (ache* or pain* or strain*)).tw.
18. lumbar vertebrae.mp.
19. (lumbago or dorsalgia).tw.
20. or/1-19
21. exp spinal manipulation/
22. exp manipulation, chiropractic/
23. chiropractic/
24. chiropract*.tw.
25. ((back or spine or spinal or lumbar or musculoskeletal) adj3 (adjust* or manipulat* or mobili*ation)).tw.
26. exp Musculoskeletal Manipulations/
27. manual therap*.tw.
28. (Manipulati* adj (therap* or medicine)).tw.
29. (Flexion adj2 distraction*).tw.
30. (myofascial adj3 (release or therap*)).tw.
31. Muscle energy technique*.tw.
32. Trigger point*.tw.
33. Trigger Points/
34. Proprioceptive Neuromuscular Facilitation.tw.
35. Cyriax Friction.tw.
36. (Strain adj counterstrain).tw.
37. (Craniosacral Therap* or Cranio sacral Therap*).tw.
38. Complementary Therapies/
39. or/21-38
40. randomised controlled trial.pt.
41. randomized controlled trial.pt.
42. clinical trial.pt.
43. random*ed.ti,ab.
44. placebo.ti,ab.
45. randomly.ti,ab.
46. trial.ti,ab.
47. groups.ti,ab.
48. or/40-47
49. exp Animals/
50. humans/
51. 49 not (49 and 50)
52. 48 not 51
53. 20 and 39 and 52
54. limit 53 to yr=”1990-Current”

**EMBASE**

**Database: Embase Classic + Embase 1947 to 2014 April 21**

1. exp backache/
2. back pain.tw.
3. backache*.tw.
4. Sciatica/
5. Sciatica.tw.
6. (spine pain or spinal pain).tw.
7. back disorder*.tw.
8. (Sciatic adj3 (Neuralgia or Bilateral)).tw.
9. ischialgia.tw.
10. ((disc* or disk*) adj3 (degener* or displace* or prolaps* or hernia* or bulge or protrusion* or extrusion* or sequestration* or disorder* or disease* or rupture* or slipped)).tw.
11. vertebral canal Stenosis/
12. ((stenosis or stenoses) adj3 (lumbar or spine or spines or spinal)).tw.
13. Diskitis/
14. (diskitis or discitis).tw.
15. spondylodiscitis.tw.
16. vertebrogenic pain syndrome*.tw.
17. ((Zygapophyseal or Facet or facets) adj3 (syndrome* or degenerat*)).tw.
18. (lumbar adj3 (ache* or pain* or strain*)).tw.
19. lumbar vertebrae.tw.
20. (lumbago or dorsalgia).tw.
21. or/1-20
22. chiropractic/
23. chiropract*.tw.
24. ((back or spine or spinal or lumbar or musculoskeletal) adj3 (adjust* or manipulat* or mobili*ation)).tw.
25. exp Musculoskeletal medicine/
26. manual therap*.tw.
27. (Manipulati* adj (therap* or medicine)).tw.
28. (Flexion adj2 distraction*).tw.
29. (myofascial adj3 (release or therap*)).tw.
30. Muscle energy technique*.tw.
31. Trigger point*.tw.
32. Trigger Points/
33. Proprioceptive Neuromuscular Facilitation.tw.
34. Cyriax Friction.tw.
35. (Strain adj counterstrain).tw.
36. (Craniosacral Therap* or Cranio sacral Therap*).tw.
37. or/22-36
38. randomized controlled trial/
39. clinical trial/
40. randomi?ed.tw.
41. placebo.tw.
42. randomly.tw.
43. trial.tw.
44. groups.tw.
45. or/38-44
46. animals/
47. humans/
48. 46 not (46 and 47)
49. 45 not 48
50. 21 and 37 and 49
51. limit 50 to yr=”1990-Current”

**CINAHL**

**Database: CINAHL Plus with Full Text (Full text from 1937-)**

S44. S13 AND S25 AND S43; Limiters: Published Date: 19900101-

S43. 39 NOT S42

S42. S40 NOT (S40 AND S41)

S41. (MH “Human”)

S40. (MH “Animals”)

S39. S26 OR S27 or S28 or S29 or S30 or S31 or S32 or S33 or S34 or S35 or S36 or S37 or S38

S38. AB groups

S37. TI groups

S36. TI trial

S35. AB trial

S34. AB randomly

S33. TI randomly

S32. TI placebo

S31. AB placebo

S30. AB randomized

S29. AB randomised

S28. TI randomised

S27. TI randomized

S26. (PT clinical trial) OR (PT randomized controlled trial)

S25. S14 OR S15 OR S16 OR S17 OR S18 OR S19 OR S20 OR S21 OR S22 OR S23 OR S24

S24. (MH “Trigger Point”)

S23. (MH “Manual Therapy”)

S22. TI ( (Strain N1 counterstrain) or “Craniosacral Therap*” or “Cranio sacral Therap*” ) OR AB ( (Strain N1 counterstrain) or “Craniosacral Therap*” or “Cranio sacral Therap*” )

S21. TI ( “muscle energy technique*” or “trigger point*” or “Proprioceptive Neuromuscular Facilitation” ) OR AB ( “muscle energy technique*” or “trigger point*” or “Proprioceptive Neuromuscular Facilitation” )

S20. TI ( (myofascial) N3 (release or therap*) ) OR AB ( (myofascial) N3 (release or therap*) )

S19. TI (Flexion N2 distraction*) OR AB (Flexion N2 distraction*)

S18. TI ( (Manipulati* N1 (therap* or medicine)) ) OR AB ( (Manipulati* N1 (therap* or medicine)) )

S17. TI “manual therap*” OR AB “manual therap*”

S16. TI ( ((back or spine or spinal or lumbar or musculoskeletal) N3 (adjust* or manipulat* or mobili*ation)) ) OR AB ( ((back or spine or spinal or lumbar or musculoskeletal) N3 (adjust* or manipulat* or mobili*ation)) )

S15. TI chiropract* OR AB chiropract*

S14. (MH “Chiropractic + ”)

S13. S1 OR S2 OR S3 OR S12

S12. S4 OR S5 OR S6 OR S7 OR S8 OR S9 OR S10 OR S11

S11. TI ( “lumbar vertebrae” or lumbago or dorsalgia ) AND AB ( “lumbar vertebrae” or lumbago or dorsalgia)

S10. TI ( ((lumbar) N3 (ache* or pain* or strain*)) ) AND AB ( ((lumbar) N3 (ache* or pain* or strain*)) )

S9. TI ( ((Zygapophyseal or Facet or facets) N3 (syndrome* or degenerat*)) ) OR AB ( ((Zygapophyseal or Facet or facets) N3 (syndrome* or degenerat*)) )

S8. TI ( spondylodiscitis OR “vertebrogenic pain syndrome* ) OR AB ( spondylodiscitis OR ”vertebrogenic pain syndrome* )

S7. TI ( ((stenosis or stenoses) N3 (lumbar or spine or spines or spinal)) ) OR AB ( ((stenosis or stenoses) N3 (lumbar or spine or spines or spinal)) )

S6. TI ( ((disc* or disk*) N3 (degener* or displace* or prolaps* or hernia* or bulge or protrusion* or extrusion* or sequestration* or disorder* or disease* or rupture* or slipped)) ) OR AB ( ((disc* or disk*) N3 (degener* or displace* or prolaps* or hernia* or bulge or protrusion* or extrusion* or sequestration* or disorder* or disease* or rupture* or slipped)) )

S5. TI ( (sciatic N3 (neuralgia or bilateral)) ) OR AB ( (sciatic N3 (neuralgia or bilateral)) )

S4. TI ( “back pain” or backache* or sciatica or “spine pain” or “spinal pain” or “back disorder*” ) OR AB ( “back pain” or backache* or sciatica or “spine pain” or “spinal pain” or “back disorder*” )

S3. (MH “Spinal Stenosis”)

S2. (MH “Sciatica”)

S1. (MH “Back Pain + ”)

**COCHRANE**

1. MeSH descriptor: [Back Pain] explode all trees
2. MeSH descriptor: [Sciatica] explode all trees
3. MeSH descriptor: [Spinal Stenosis] explode all trees
4. MeSH descriptor: [Discitis] explode all trees
5. back near pain:ti,ab,kw
6. backache* or sciatica or spine near pain or spinal near pain or back near disorder*:ti,ab,kw
7. (sciatic near/3 (neuralgia or bilateral)) or ((disc* or disk*) near/3 (degener* or displace* or prolaps* or hernia* or bulge or protrusion* or extrusion* or sequestration* or disorder* or disease* or rupture* or slipped))
8. ((stenosis or stenoses) near/3 (lumbar or spine or spines or spinal)) or spondylodiscitis or ((Zygapophyseal or Facet or facets) near/3 (syndrome* or degenerat*)) or ((lumbar) near/3 (ache* or pain* or strain*)) or “lumbar vertebrae” or lumbago or dorsalgia
9. #1 or #2 or #3 or #4 or #5 or #6 or #7 or #8
10. MeSH descriptor: [Manipulation, Spinal] explode all trees
11. MeSH descriptor: [Manipulation, Chiropractic] explode all trees
12. MeSH descriptor: [Chiropractic] explode all trees
13. MeSH descriptor: [Musculoskeletal Manipulations] this term only
14. MeSH descriptor: [Trigger Points] explode all trees
15. MeSH descriptor: [Complementary Therapies] this term only
16. chiropract* or ((back or spine or spinal or lumbar or musculoskeletal) near/3 (adjust* or manipulat* or mobili*ation)) or manual near therap* or (Manipulati* near (therap* or medicine)) or (Flexion near/2 distraction*) or (myofascial near/3 (release or therap*)) or trigger near point* or (Strain near counterstrain) or Craniosacral near Therap*:ti,ab,kw
17. #10 or #11 or #12 or #13 or #14 or #15 or #16
18. #9 and #17

**Note: Only include results from Cochrane Central (Trials); publication date 1990-**

**Appendix 2: Search strategy for the review of economic evaluation**

MEDLINE

1. exp Back Pain/
2. back pain.tw.
3. backache*.tw.
4. Sciatica/
5. Sciatica.tw.
6. (spine pain or spinal pain).tw.
7. back disorder*.tw.
8. (Sciatic adj3 (Neuralgia or Bilateral)).ti,ab.
9. ischialgia.tw.
10. ((disc* or disk*) adj3 (degener* or displace* or prolaps* or hernia* or bulge or protrusion* or extrusion* or sequestration* or disorder* or disease* or rupture* or slipped)).tw.
11. Spinal Stenosis/
12. ((stenosis or stenoses) adj3 (lumbar or spine or spines or spinal)).tw.
13. Discitis/
14. diskitis.ti,ab.
15. spondylodiscitis.ti,ab.
16. vertebrogenic pain syndrome*.tw.
17. ((Zygapophyseal or Facet or facets) adj3 (syndrome* or degenerat*)).tw.
18. (lumbar adj3 (ache* or pain* or strain*)).tw.
19. lumbar vertebrae.mp.
20. (lumbago or dorsalgia).tw.
21. 1 or 2 or 3 or 4 or 5 or 6 or 7 or 8 or 9 or 10 or 11 or 12 or 13 or 14 or 15 or 16 or 17 or 18 or 19 or 20
22. exp spinal manipulation/
23. manipulation, chiropractic/
24. chiropractic/
25. chiropract*.tw.
26. ((back or spine or spinal or lumbar or musculoskeletal) adj3 (adjust* or manipulat* or mobili*ation)).tw.
27. exp Musculoskeletal Manipulations/
28. manual therap*.tw.
29. (Manipulati* adj (therap* or medicine)).tw.
30. (Flexion adj2 distraction*).tw.
31. (myofascial adj3 (release or therap*)).tw.
32. Muscle energy technique*.tw.
33. Trigger point*.tw.
34. Trigger Points/
35. Proprioceptive Neuromuscular Facilitation.tw.
36. Cyriax Friction.tw.
37. (Strain adj counterstrain).tw.
38. (Craniosacral Therap* or Cranio sacral Therap*).tw.
39. Complementary Therapies/
40. or/22-39
41. economics/
42. exp “Costs and Cost Analysis”/
43. “Value of Life”/
44. resource allocation/ or exp economics, medical/ or “fees and charges”/
45. (econom* or cost* or pric* or fee or fees or expense* or saving* or financial or “loss reduction” or payback* or “return on investment”).tw.
46. (benefit* or “wage replacement”).tw.
47. budget.ti,ab.
48. (resource* adj2 allocation).tw.
49. economics.fs.
50. or/41-49
51. 21 and 40 and 50

EMBASE

1. exp backache/
2. back pain.tw.
3. backache*.tw.
4. Sciatica/
5. Sciatica.tw.
6. (spine pain or spinal pain).tw.
7. back disorder*.tw.
8. (Sciatic adj3 (Neuralgia or Bilateral)).ti,ab.
9. ischialgia.tw.
10. ((disc* or disk*) adj3 (degener* or displace* or prolaps* or hernia* or bulge or protrusion* or extrusion* or sequestration* or disorder* or disease* or rupture* or slipped)).tw.
11. vertebral canal Stenosis/
12. ((stenosis or stenoses) adj3 (lumbar or spine or spines or spinal)).tw.
13. Diskitis/
14. (diskitis or discitis).ti,ab.
15. spondylodiscitis.ti,ab.
16. vertebrogenic pain syndrome*.tw.
17. ((Zygapophyseal or Facet or facets) adj3 (syndrome* or degenerat*)).tw.
18. (lumbar adj3 (ache* or pain* or strain*)).tw.
19. lumbar vertebrae.mp.
20. (lumbago or dorsalgia).tw.
21. 1 or 2 or 3 or 4 or 5 or 6 or 7 or 8 or 9 or 10 or 11 or 12 or 13 or 14 or 15 or 16 or 17 or 18 or 19 or 20
22. chiropractic/
23. chiropract*.tw.
24. ((back or spine or spinal or lumbar or musculoskeletal) adj3 (adjust* or manipulat* or mobili*ation)).tw.
25. exp Musculoskeletal medicine/
26. manual therap*.tw.
27. (Manipulati* adj (therap* or medicine)).tw.
28. (Flexion adj2 distraction*).tw.
29. (myofascial adj3 (release or therap*)).tw.
30. Muscle energy technique*.tw.
31. Trigger point*.tw.
32. Trigger Points/
33. Proprioceptive Neuromuscular Facilitation.tw.
34. Cyriax Friction.tw.
35. (Strain adj counterstrain).tw.
36. (Craniosacral Therap* or Cranio sacral Therap*).tw.
37. economics/
38. exp economic evaluation/
39. resource allocation/ or health economics/ or medical fee/
40. (econom* or cost* or pric* or fee or fees or expense* or saving* or financial or “loss reduction” or payback* or “return on investment”).tw.
41. (benefit* or “wage replacement”).tw.
42. budget.ti,ab.
43. (resource* adj2 allocation).tw.
44. or/22-36
45. or/37-43
46. 21 and 44 and 45

AMED

1. exp backache/
2. back pain.tw.
3. backache*.tw.
4. Sciatica/
5. Sciatica.tw.
6. (spine pain or spinal pain).tw.
7. back disorder*.tw.
8. (Sciatic adj3 (Neuralgia or Bilateral)).ti,ab.
9. ischialgia.tw.
10. ((disc* or disk*) adj3 (degener* or displace* or prolaps* or hernia* or bulge or protrusion* or extrusion* or sequestration* or disorder* or disease* or rupture* or slipped)).tw.
11. Spinal Stenosis/
12. ((stenosis or stenoses) adj3 (lumbar or spine or spines or spinal)).tw.
13. diskitis.ti,ab.
14. spondylodiscitis.ti,ab.
15. vertebrogenic pain syndrome*.tw.
16. ((Zygapophyseal or Facet or facets) adj3 (syndrome* or degenerat*)).tw.
17. (lumbar adj3 (ache* or pain* or strain*)).tw.
18. lumbar vertebrae.mp.
19. (lumbago or dorsalgia).tw.
20. exp spinal manipulation/
21. exp manipulation, chiropractic/
22. chiropractic/
23. chiropract*.tw.
24. ((back or spine or spinal or lumbar or musculoskeletal) adj3 (adjust* or manipulat* or mobili*ation)).tw.
25. exp Musculoskeletal Manipulations/
26. manual therap*.tw.
27. (Manipulati* adj (therap* or medicine)).tw.
28. (Flexion adj2 distraction*).tw.
29. (myofascial adj3 (release or therap*)).tw.
30. Muscle energy technique*.tw.
31. Trigger point*.tw.
32. Trigger Points/
33. Proprioceptive Neuromuscular Facilitation.tw.
34. Cyriax Friction.tw.
35. (Strain adj counterstrain).tw.
36. (Craniosacral Therap* or Cranio sacral Therap*).tw.
37. Complementary Therapies/
38. or/20-37
39. economics/
40. exp “Costs and Cost Analysis”/
41. “fees and charges”/
42. (econom* or cost* or pric* or fee or fees or expense* or saving* or financial or “loss reduction” or payback* or “return on investment”).tw.
43. (benefit* or “wage replacement”).tw.
44. budget.ti,ab.
45. (resource* adj2 allocation).tw.
46. or/1-19
47. or/39-45
48. 38 and 46 and 47

CINAHL and Cochrane

Almost identical to MEDLINE

## Additional file

Additional file 1:
**Data extraction form for economic evaluation.**

## Abbreviations

BMJ: British medical journal
CADTH: Canadian agency for drugs and technologies in health
CIHI: Canadian institute for health information
CIHR: Canadian institute of health research
CPGs: Clinical practice guidelines
GDP: Gross domestic product
ICL: Index to chiropractic literature
LBP: Low back pain
NICE: National Institute for health and care excellence
PPP: Purchasing power parities
QALYs: Quality-adjusted life years
SMT: Spinal manipulative therapy

## References

[1] Vos T, Flaxman AD, Naghavi M, Lozano R, Michaud C, Ezzati M (2012). Years lived with disability (YLDs) for 1160 sequelae of 289 diseases and injuries 1990–2010: a systematic analysis for the Global Burden of Disease Study 2010. Lancet.

[2] Deyo RA, Mirza SK, Martin BI (2006). Back pain prevalence and visit rates: estimates from U.S. national surveys, 2002. Spine (Phila Pa 1976).

[3] Leroux I, Dionne CE, Bourbonnais R, Brisson C (2005). Prevalence of musculoskeletal pain and associated factors in the Quebec working population. Int Arch Occup Environ Health.

[4] Druss BG, Marcus SC, Olfson M, Pincus HA (2002). The most expensive medical conditions in America. Health Aff (Millwood).

[5] Hashemi L, Webster BS, Clancy EA (1998). Trends in disability duration and cost of workers’ compensation low back pain claims (1988–1996). J Occup Environ Med.

[6] Mattila VM, Sillanpaa P, Visuri T, Pihlajamaki H (2009). Incidence and trends of low back pain hospitalisation during military service–an analysis of 387,070 Finnish young males. BMC Musculoskelet Disord.

[7] Murphy PL, Volinn E (1999). Is occupational low back pain on the rise?. Spine (Phila Pa 1976).

[8] Waddell G (2004). The Back Pain Revolution.

[9] Hoy D, March L, Brooks P, Blyth F, Woolf A, Bain C (2014). The global burden of low back pain: estimates from the Global Burden of Disease 2010 study. Ann Rheumatic Dis.

[10] Hayden JA, van Tulder MW, Malmivaara A, Koes BW (2005). Exercise therapy for treatment of non-specific low back pain. Cochrane Database Syst Rev.

[11] Rubinstein SM, van Middelkoop M, Assendelft WJ, de Boer MR, van Tulder MW (2011). Spinal manipulative therapy for chronic low-back pain: an update of a Cochrane review. Spine (Phila Pa 1976).

[12] van Oostrom SH, Driessen MT, de Vet HC, Franche RL, Schonstein E, Loisel P (2009). Workplace interventions for preventing work disability. Cochrane Database Syst Rev.

[13] Chou R, Atlas SJ, Stanos SP, Rosenquist RW (2009). Nonsurgical interventional therapies for low back pain: a review of the evidence for an American Pain Society clinical practice guideline. Spine (Phila Pa 1976).

[14] Chou R, Loeser JD, Owens DK, Rosenquist RW, Atlas SJ, Baisden J (2009). Interventional therapies, surgery, and interdisciplinary rehabilitation for low back pain: an evidence-based clinical practice guideline from the American Pain Society. Spine (Phila Pa 1976).

[15] Haldeman S, Dagenais S (2008). A supermarket approach to the evidence-informed management of chronic low back pain. Spine J.

[16] Chou R, Qaseem A, Snow V, Casey D, Cross JT, Shekelle P (2007). Diagnosis and treatment of low back pain: a joint clinical practice guideline from the American College of Physicians and the American Pain Society. Ann Internal Med.

[17] Haldeman S, Kopansky-Giles D, Hurwitz EL, Hoy D, Erwin MW, Dagenais S (2012). Advancements in the management of spine disorders. Best Pract Res Clin Rheumatol.

[18] Haldeman S, Carroll LJ, Cassidy JD (2008). The empowerment of people with neck pain: introduction: the Bone and Joint Decade 2000–2010 Task Force on Neck Pain and Its Associated Disorders. Spine (Phila Pa 1976).

[19] Rubinstein SM, Terwee CB, Assendelft WJ, de Boer MR, van Tulder MW (2012). Spinal manipulative therapy for acute low-back pain. Cochrane Database Syst Rev.

[20] Rubinstein SM, Terwee CB, Assendelft WJ, de Boer MR, van Tulder MW (2013). Spinal manipulative therapy for acute low back pain: an update of the Cochrane review. Spine (Phila Pa 1976).

[21] Shekelle PG, Adams AH, Chassin MR, Hurwitz EL, Brook RH (1992). Spinal manipulation for low-back pain. Ann Internal Med.

[22] Haas M, Sharma R, Stano M (2005). Cost-effectiveness of medical and chiropractic care for acute and chronic low back pain. J Manipulative Physiol Ther.

[23] Manga P, Angus DE (1998). Enhanced chiropractic coverage under OHIP as a means of reducing health care costs, attaining better health outcomes and achieving equitable access to select health services.

[24] Manga P, Angus D, Papadopoulos C, Swan W (1993). The effectiveness and cost-effectiveness of chiropractic management of low-back pain.

[25] Brown A, Angus D, Chen S, Tang Z, Milne S, Pfaff J et al. Costs and outcomes of chiropractic treatment for low back pain (structured abstract). Health Technol Assess Database*.* 2005(2):88. http://www.cadth.ca/en/products/health-technology-assessment/publication/525.

[26] Dagenais S, Tricco AC, Haldeman S (2010). Synthesis of recommendations for the assessment and management of low back pain from recent clinical practice guidelines. Spine J.

[27] Baldwin ML, Cote P, Frank JW, Johnson WG (2001). Cost-effectiveness studies of medical and chiropractic care for occupational low back pain. A critical review of the literature. Spine J.

[28] Palmer S, Torgerson DJ (1999). Economic notes: definitions of efficiency. BMJ.

[29] Drummond MF, Sculpher M, Torrance G, O’Brien B, Stoddart G (2005). Methods for the economic evaluation of health care programmes.

[30] Briggs AH, O’Brien BJ (2001). The death of cost-minimization analysis?. Health Econ.

[31] Higgins JP, Green S (2008). Cochrane handbook for systematic reviews of interventions.

[32] Furlan AD, Pennick V, Bombardier C, van Tulder M (2009). 2009 updated method guidelines for systematic reviews in the Cochrane Back Review Group. Spine (Phila Pa 1976).

[33] Tompa E, Culyer AJ, Dolinschi R (2008). Economic Evaluation of Interventions for Occupational Health and Safety Developing Good Practice.

[34] Drummond MF, Jefferson TO (1996). Guidelines for authors and peer reviewers of economic submissions to the BMJ. The BMJ Economic Evaluation Working Party. BMJ.

[35] Group CBR. http://back.cochrane.org/forms. Accessed June 20th 2014.

[36] Slavin RE (1995). Best evidence synthesis: an intelligent alternative to meta-analysis. J Clin Epidemiol.

[37] Tompa E, Verbeek J, van Tulder M, de Boer A (2010). Developing guidelines for good practice in the economic evaluation of occupational safety and health interventions. Scand J Work Environ Health.

[38] Tompa E, Dolinschi R, de Oliveira C, Amick BC, Irvin E (2010). A systematic review of workplace ergonomic interventions with economic analyses. J Occup Rehabil.

[39] Tompa E, Dolinschi R, de Oliveira C, Irvin E (2009). A systematic review of occupational health and safety interventions with economic analyses. J Occup Environ Med.

[40] Moher D, Liberati A, Tetzlaff J, Altman DG (2009). Preferred reporting items for systematic reviews and meta-analyses: the PRISMA statement. BMJ.

