# Supplementary material for: Effectiveness and economic evaluation of chiropractic care for the treatment of low back pain: a systematic review protocol
Source: Syst Rev. 2015 Mar 18;4:30. doi: 10.1186/s13643-015-0015-5 (PMC4369074; doi:10.1186/s13643-015-0015-5)
Supplement: Additional file 1: — Data extraction form for economic evaluation. [file 13643_2015_15_MOESM1_ESM.doc]

**Additional file 1: Data extraction form for economic evaluation**

| **First Author, Year, Country,**  **Type of economic evaluation** | **Participants,  Indication and Setting** | **Compared Treatments** | **Perspective,**  **Time Horizon, Currency Price (Year)** | **Included Costs,**  **Health Effect**  **(**pain, functional status, global improvement, health related quality of life, return to work**)** | **Mean Health effect,**  **Mean Costs, Mean QALYs,**  **Incremental Cost-effectiveness Statistics, Incremental net-benefit, limitations, Authors’ Conclusion** |
| --- | --- | --- | --- | --- | --- |
|  |  |  |  |  |  |
